# Intrinsically Bayesian robust classifier for single-cell gene expression trajectories in gene regulatory networks

**DOI:** 10.1186/s12918-018-0549-y

**Published:** 2018-03-21

**Authors:** Alireza Karbalayghareh, Ulisses Braga-Neto, Edward R. Dougherty

**Affiliations:** 1 Department of Electrical and Computer Engineering, Texas A&M University, College Station, 77843 TX USA; 2 Center for Bioinformatics and Genomic Systems Engineering, College Station, 77843 TX USA

**Keywords:** Intrinsically Bayesian robust classifier, Optimal Bayesian classifier, Bayesian trajectory classifier, Single-cell expression trajectory, Gene regulatory network, Boolean network, Bayesian partially observed Boolean dynamical system

## Abstract

**Background:**

Expression-based phenotype classification using either microarray or RNA-Seq measurements suffers from a lack of specificity because pathway timing is not revealed and expressions are averaged across groups of cells. This paper studies expression-based classification under the assumption that single-cell measurements are sampled at a sufficient rate to detect regulatory timing. Thus, observations are expression trajectories. In effect, classification is performed on data generated by an underlying gene regulatory network.

**Results:**

Network regulation is modeled via a Boolean network with perturbation, regulation not fully determined owing to inherent biological randomness. The binary assumption is not critical because the resulting Markov chain characterizes expression trajectories. We assume a partially known Gaussian observation model belonging to an uncertainty class of models. We derive the intrinsically Bayesian robust classifier to discriminate between wild-type and mutated networks based on expression trajectories. The classifier minimizes the expected error across the uncertainty class relative to the prior distribution. We test it using a mammalian cell-cycle model, discriminating between the normal network and one in which gene p27 is mutated, thereby producing a cancerous phenotype. Tests examine all model aspects, including trajectory length, perturbation probability, and the hyperparameters governing the prior distribution over the uncertainty class.

**Conclusions:**

Simulations show the rates at which the expected error is diminished by smaller perturbation probability, longer trajectories, and hyperparameters that tighten the prior distribution relative to the unknown true network. For average-expression measurement, methods have been proposed to obtain prior distributions. These should be extended to the more mathematically difficult, but more informative, expression trajectories.

**Electronic supplementary material:**

The online version of this article (10.1186/s12918-018-0549-y) contains supplementary material, which is available to authorized users.

## Background

Phenotype classification is a salient issue for translational genomics, for instance, classification of normal versus cancerous cells, of different stages of tumor development, or different prospective drug response. Both expression microarray and RNA-Seq measurements have received much interest; however, they suffer from a lack of specificity. First, pathway timing is not reflected in the data and, second, expressions are averaged across groups of cells, so that individual cell responses are not detectable. New technologies are being developed for profiling individual cells using RNA-Seq or quantitative PCR [1]. Individual cells can be captured via standard methods, such as flow cytometry, glass capillaries, or laser [2], and be measured at various time points.

Genes have interactions with each other, which can determine how they are behaving over time and define the dynamics of gene regulatory networks (GRNs). One way of showing the dynamics of GRNs over discrete time points is Boolean networks with perturbation (BNp) [3]. A BNp is a Markov chain, in which the state of a gene (0 for off and 1 for on) at the current time is a function of the states of its predictor genes at the previous time plus a small random Boolean noise. Network inference could be done using RNA-Seq time series measurements [4].

Suppose we have single-cell measurements sampled with a sufficient rate to detect regulatory timing. In effect, this would mean that classification would be done on data reflecting an underlying gene regulatory network. In [5] we proposed a classifier to classify the state trajectories of the two classes: wild-type and mutated, each having its own BNp. We derived the Bayes classifier and computed the Bayes error. We analyzed the effects of the length of the trajectories, perturbation probability, and different mutations on the Bayes error.

In [6] and [7] we assumed an observation model on the state dynamics of the BNps, from which the expression values of the genes are obtained. As the parameters of the model were all unknown and the network functions were partially known, we proposed an expectation-maximization (EM)-based algorithm to estimate these parameters and functions, and then plugged them into the Bayes classifier. In [6] we assumed that the expression trajectory data come from single-cell measurements and compared that with a multiple-cell scenario, in which instead of trajectories we have the averaged expressions of all genes over all cells, which translates to an average over all states.

In this paper we extend the single-cell trajectory classification to the Bayesian framework. We propose the intrinsically Bayesian robust (IBR) classifier for the trajectorires. The IBR classifier is a specific type of the obtimal Bayesian classifier (OBC), first introduced in [8, 9] for the classification of static data. In fact, the difference between the OBC and IBR classifiers is that in the OBC the expectation of the class-conditional densities is taken over the posteriors of the parameters to obtain the effective class-conditional densities, whereas in the IBR classifier the expectation is taken over the priors. The IBR/OBC concept has been applied to linear and morphological filtering [10, 11], and IBR Kalman filtering [12]. Regarding the prior distributions, prior construction methods using the pathway knowledge have been studied in the literature, such as [13, 14]. Bayesian methods for finding differentially expressed genes can be used for the aim of classification [15].

Here we apply the IBR classifier to the classification of trajectories. As opposed to [6, 7], where we estimate the parameters, here we assume that the parameters belong to an uncertainty class governed by a prior distribution. We assume that there are two classes: wild-type (*S*=0) and mutated (*S*=1). We introduce a Bayesian version of the partially observed Boolean dynamical system (POBDS), proposed in [16], as the observation model. We use a beta prior distribution for the prior probability of the class *S*=0 and also for the gene perturbation probability. Since the observation model given the states is Gaussian, we employ the normal-gamma distribution as the prior distribution of the mean and precision (inverse of variance) of the Gaussian model.

In the simulation part, we employ a mammalian cell-cycle gene regulatory network [17] consisting of 10 genes as the BNp for the class *S*=0 and its mutated version as the BNp for the class *S*=1. We analyze the effects of all the hyperparameters of the model and the length of the observed trajectories on the classification error. The proposed classifier is computationally efficient because we use the sum-product method to reduce the complexity to *m*×2^*n*^, where *m* is the length of the observed trajectory and *n* is the number of genes in the network. Being linearly dependent on time points, *m*, makes the classifier very fast even for longer trajectories.

## Methods

In a Boolean network (BN) for *n* genes, each gene value *x*_*i*_∈{0,1}, for *i*=1,⋯,*n*, at time *k*+1 is determined by the values of some predictor genes at time *k* via a Boolean function *f*_*i*_:{0,1}^*n*^→{0,1}. In practice, *f*_*i*_ is a function of a small number of genes, *K*_*i*_, called the *in-degree* of the gene *x*_*i*_ in the network. The in-degree of the network is *K*= max*i*=1,⋯,*nK*_*i*_. A gene network can be represented as a graph with vertices representing genes and edges representing regulations. There is a state diagram of 2^*n*^ states corresponding to the truth table of the BN, representing the dynamics of the network. Given an initial state, a BN will eventually reach a set of states, called an *attractor cycle*, through which it will cycle endlessly. Each initial state corresponds to a unique attractor cycle, and the set of initial states leading to a specific attractor cycle is known as the *basin of attraction* (BOA) of the attractor cycle.

### State model

We allow stochasticity in our state model by using Boolean networks with perturbation (BNps) instead of deterministic BNs. For BNps, perturbation is introduced with a probability *p*_*k*_ by which the state of a gene in the network can be randomly flipped at time *k*. We assume that there is an independent identically distributed (i.i.d.) random perturbation vector at each time *k*, denoted by **n**_*k*_∈{0,1}^*n*^, such that the *i*-th gene flips at time *k* if the *i*-th component of **n**_*k*_ is equal to 1. Therefore, the dynamical model can be expressed as

1 $$ \mathbf{X}_{k+1}=\mathbf{f}(\mathbf{X}_{k})\oplus \mathbf{n}_{k+1}, \quad k=0,1,2,\cdots,  $$

where **X**_*k*_=[*x*_1_(*k*),*x*_2_(*k*),⋯,*x*_*n*_(*k*)]^*T*^ is a binary state vector, called a *gene activity profile* (GAP), at time *k*, in which *x*_*i*_(*k*) indicates the expression level of the *i*-th gene at time *k* (either 0 or 1); **f**=[*f*_1_,*f*_2_,⋯,*f*_*n*_]^*T*^:{0,1}^*n*^→{0,1}^*n*^ is the vector of the network functions, in which *f*_*i*_ shows the expression level of the *i*-th gene at time *k*+1 when the system lies in the state **X**_*k*_ at time *k*; **n**_*k*_=[*n*_1_(*k*),*n*_2_(*k*),⋯,*n*_*n*_(*k*)]^*T*^ is the perturbation vector at time *k*, in which *n*_1_(*k*),*n*_2_(*k*),⋯,*n*_*n*_(*k*) are i.i.d. Bernoulli random variables for every *k* with the parameter *p*_*k*_=*P*(*n*_*i*_(*k*)=1) for *i*=1,⋯,*n*; and ⊕ is component-wise modulo 2 addition.

The existence of perturbation makes the corresponding Markov chain of a BNp irreducible. Hence, the network possesses a steady-state distribution *π* describing its long-run behavior. If *p*_*k*_ is sufficiently small, *π* will reflect the attractor structure within the original network. We can derive the transition probability matrix (TPM) if we know the truth table and the perturbation probability of a BNp. As a result, the steady-state distribution *π* can be computed as well.

#### Prior for state parameter

In this paper, we assume that we know the underlying Boolean networks for both the wild-type and mutated classes, and the only uncertain parameter at the state level is the perturbation probability *p*_*k*_. Since 0<*p*_*k*_<1, we can employ the beta prior for *p*_*k*_, for all *k*=1,2,⋯, as

2 $$ g(p_{k}) \sim \text{Beta}(a,b) = \frac{\Gamma(a+b)}{\Gamma(a)\Gamma(b)} p_{k}^{a-1} (1-p_{k})^{b-1},  $$

where *a* and *b* are known parameters. Since in reality *p*_*k*_ is close to zero, we can choose *a* and *b* in such a way that this fact is satisfied. To this end, we can use the mean and variance of *p*_*k*_:

3 $$ \mathrm{E}\left[p_{k}\right]=\frac{a}{a+b}, \quad \text{Var}\left[p_{k}\right] = \frac{ab}{(a+b)^{2}(a+b+1)}.  $$

### Observation model

We define a Bayesian partially-observed Boolean dynamical system (BPOBDS) as the model for the gene expression data. In this model, we assume that the gene expressions come from Gaussian distributions whose parameters are governed by prior distributions whose parameters (the hyperparameters of the observations) are a function of the hidden Boolean states. If *y*_*j*_(*k*) is the expression value of the *j*-th gene at time *k*, then the observation model is

4 $$ p\left(y_{j}(k)|\theta_{j}(k),\lambda_{j}(k)\right) \sim \mathcal{N}\left(\theta_{j}(k),\lambda_{j}(k)^{-1}\right),  $$

for *j*=1,2,⋯,*n* and *k*=1,2,⋯, where *θ*_*j*_(*k*) and *λ*_*j*_(*k*) denote the mean and precision, respectively, of the Gaussian distribution.

#### Priors for observation parameters

We employ the well-known normal-gamma prior distribution for *θ*_*j*_(*k*) and *λ*_*j*_(*k*):

5 $$\begin{array}{@{}rcl@{}} p(\lambda_{j}(k)) &\sim& \text{Gamma}(\alpha_{0},\beta_{0}),  \\ p(\theta_{j}(k)| \lambda_{j}(k), x_{j}(k)) &\sim& \mathcal{N} \left(\mu_{j}(k),(\kappa_{0} \lambda_{j}(k))^{-1}\right),  \\ \text{where} ~~~ \mu_{j}(k) &=& \mu_{0} + \delta_{0} x_{j}(k), \end{array} $$

where *α*_0_, *β*_0_, *κ*_0_, *μ*_0_, and *δ*_0_ are known positive hyperparameters, and *x*_*j*_(*k*) is the hidden Boolean state of gene *j* at time *k*. The intuition behind the prior (5) is that when gene *j* at time *k* is on or off, that is, *x*_*j*_(*k*)=1 or 0, the hyper-mean of the expression for that gene is *μ*_*j*_(*k*)=*μ*_0_+*δ*_0_ or *μ*_*j*_(*k*)=*μ*_0_, respectively, at time *k*. In (5), *μ*_0_ is the baseline expression level and *δ*_0_ is the expression coefficient. The hyperparameters *α*_0_, *β*_0_, and *κ*_0_ determine the level of uncertainty, by which we can control the variance of the outputs. We assume the same values of hyperparameters for all genes at all times.

### IBR classifier

If one knows the feature-label distribution, then the error of any classifier can be found and an optimal (Bayes) classifier minimizes classifier error. If the feature-label distribution is unknown but belongs to an uncertainty class *Θ* of feature-label distributions, then we desire a classifier to minimize the expected error over the uncertainty class. Given a classifier *ψ*, from the perspective of mean-square error (MSE), the best error estimate minimizes the MSE between its true error (a function of parameter **θ**) and an error estimate. This Bayesian minimum-mean-square-error (MMSE) estimate is given by the expected true error, $\widehat {\varepsilon }(\psi)=\mathrm {E}_{\theta }\left [\varepsilon (\psi,\mathbf {\theta })\right ]$, where *ε*(*ψ*,**θ**) is the error of *ψ* on the feature-label distribution parameterized by **θ** and the expectation is taken relative to the prior distribution *π*(*θ*) [18].

An IBR classifier minimizes the Bayesian MMSE estimate. If *ψ*(**x**)=0 if **x**∈*R*_0_ and *ψ*(**x**)=1 if **x**∈*R*_1_, where **x** is a multidimensional vector of data, and *R*_0_ and *R*_1_ partition the sample space, then [8]

$${} \widehat{\varepsilon }\left(\psi \right) =\mathrm{E}_{\pi}[\!c]\int_{R_{1}}f_{\Theta}\left(\mathbf{x}|0\right) d\mathbf{x}+\left(1-\mathrm{E}_{\pi }[\!c]\right)\int_{R_{0}}f_{\Theta}\left(\mathbf{x}|1\right) d\mathbf{x}, $$

where

$$f_{\Theta}\left(\mathbf{x}|y\right) =\int_{\mathbf{\Theta }_{y}}f_{\mathbf{\theta}_{y}}\left(\mathbf{x}|y\right) \pi \left(\mathbf{\theta}_{y}\right) d\mathbf{\theta }_{y} $$

is the *effective class-conditional density* for class *y*, **Θ**_*y*_ being the space for **θ**_*y*_, $f_{\mathbf {\theta }_{y}}\left (\mathbf {x}|y\right)$ is the class-conditional density, and *c* is the prior probability of the class 0. The IBR classifier is given by [8]

6 $$ \psi_{\text{IBR}}\left(\mathbf{x}\right) =\left\{ \begin{array}{ll} 0 \quad \text{if ~~ }\mathrm{E}_{\pi }[\!c]f_{\Theta}\left(\mathbf{x}|0\right) \geq \\ \qquad \quad \left(1-\mathrm{E}_{\pi }[\!c]\right)f_{\Theta}\left(\mathbf{x}|1\right) & \\ 1 \quad \text{otherwise} \end{array} \right..  $$

### Trajectory-based IBR classifier

Let *Θ*_*s*_=[*p*_2:*m*_,*θ*_1:*n*_(1:*m*),*λ*_1:*n*_(1:*m*)] denote the parameters of the class *S*=*s*, for *s*=0,1, where *p*_2:*m*_ means the parameters *p*_2_,*p*_3_,⋯,*p*_*m*_, and similarly for *θ*_1:*n*_(1:*m*) and *λ*_1:*n*_(1:*m*). Furthermore, let **f**_*s*_ denote the Boolean network function of the class *S*=*s* and $\mathcal {X}=\left [\mathbf {X}_{1},\mathbf {X}_{2},\cdots,\mathbf {X}_{m}\right ]$ denote the Boolean state trajectory at *m* consecutive times points at which $\mathcal {Y}$ has been observed. The *n*×1 Boolean vector **X**_*k*_=[*x*_1_(*k*),*x*_2_(*k*),⋯,*x*_*n*_(*k*)]^*T*^ has the states of the *n* genes at time *k*, which are hidden and not observed.

Suppose that we obtain the expressions of *n* genes at *m* consecutive time points. Let **Y**_*k*_=[*y*_1_(*k*),⋯,*y*_*n*_(*k*)]^*T*^ denote the *n*×1 expression vector of *n* genes at time *k*, and $\mathcal {Y}=\left [\mathbf {Y}_{1},\cdots,\mathbf {Y}_{m}\right ]$ denote a time trajectory of length *m*, containing the expression vectors at the *m* consecutive times. The problem is to optimally classify this observed trajectory $\mathcal {Y}$ to the class 0 (wild-type) or class 1 (mutated). Let *c* and 1−*c* be the prior probabilities of the class *S*=0 and class *S*=1, respectively. Since we are uncertain about *c*, we use a beta prior

7 $$ g(c) \sim \text{Beta}\left(a_{c},b_{c}\right) = \frac{\Gamma\left(a_{c}+b_{c}\right)}{\Gamma(a_{c})\Gamma(b_{c})} c^{a_{c}-1} (1-c)^{b_{c}-1},  $$

with mean

8 $$ \mathrm{E}[\!c]=\frac{a_{c}}{a_{c}+b_{c}},  $$

where *a*_*c*_ and *b*_*c*_ are known parameters.

According to (6), the IBR classifier for the trajectories is

9 $$ \psi_{\text{IBR}}(\mathcal{Y})=\left\{ \begin{array}{ll} 0 \quad \text{if} ~~ \mathrm{E}[\!c]p(\mathcal{Y}|S=0)\geq \\ \hspace{1cm} (1-\mathrm{E}[\!c])p(\mathcal{Y}|S=1) & \\ 1 \quad \text{otherwise} \end{array},\right.  $$

where $p(\mathcal {Y}|S=s)$ is the effective class-conditional density of the trajectory $\mathcal {Y}$ in the class *S*=*s* for *s*=0,1.

#### Effective class-conditional densities of trajectories

The joint distribution of $\mathcal {Y}$, $\mathcal {X}$, and *Θ*_*s*_ given the class *S*=*s* can be factorized as

10 $$ \begin{aligned} p\left(\mathcal{Y},\mathcal{X},\Theta_{s} | S=s\right) &= g(p_{2:m}) P\left(\mathcal{X}|p_{2:m}, S=s\right) \\ & \quad \times p\left(\mathcal{Y}| \theta_{1:n}(1:m),\lambda_{1:n}(1:m)\right) \\ & \quad \times p\left(\theta_{1:n}(1:m), \lambda_{1:n}(1:m)|\mathcal{X}\right). \end{aligned}  $$

In deriving (10), it is assumed that the state parameters {*p*_2:*m*_} are independent of the observation parameters {*θ*_1:*n*_(1:*m*),*λ*_1:*n*_(1:*m*)}. Due to the independence assumption in the priors,

11 $$ \begin{aligned} g(p_{2:m}) &= \prod_{k=1}^{m-1} g(p_{k+1}) \\ &= \prod_{k=1}^{m-1} \frac{\Gamma(a+b)}{\Gamma(a)\Gamma(b)} p_{k+1}^{a-1} (1-p_{k+1})^{b-1}, \end{aligned}  $$

12 $$ \begin{aligned} &p\left(\theta_{1:n}(1:m), \lambda_{1:n}(1:m)|\mathcal{X}\right) \\ &= \prod_{k=1}^{m} \prod_{j=1}^{n} p(\theta_{j}(k) | \lambda_{j}(k),x_{j}(k)) p(\lambda_{j}(k))\\ &= \prod_{k=1}^{m} \prod_{j=1}^{n} \frac{1}{\sqrt{2\pi (\kappa_{0} \lambda_{j}(k))^{-1}}} \frac{\beta_{0}^{\alpha_{0}}}{\Gamma(\alpha_{0})} \\ & \times \exp \left(-\frac{(\theta_{j}(k)-\mu_{j}(k))^{2}}{2(\kappa_{0} \lambda_{j}(k))^{-1}}\right) \lambda_{j}(k)^{\alpha_{0}-1} \exp(-\beta_{0} \lambda_{j}(k)). \end{aligned}  $$

If we assume that the conditional expressions, given the parameters, of the *n* genes at *m* time points are independent, the likelihood of $\mathcal {Y}$ given the parameters in (10) can be written as

13 $$ \begin{aligned} &p\left(\mathcal{Y}| \theta_{1:n}(1:m),\lambda_{1:n}(1:m)\right) \\ &= \prod_{k=1}^{m}\prod_{j=1}^{n} p\left(y_{j}(k) | \theta_{j}(k),\lambda_{j}(k)\right)\\ &= \prod_{k=1}^{m}\prod_{j=1}^{n} \frac{1}{\sqrt{2\pi \lambda_{j}(k)^{-1}}} \exp \left(-\frac{(y_{j}(k)-\theta_{j}(k))^{2}} {2 \lambda_{j}(k)^{-1}} \right). \end{aligned}  $$

We should note that the independency assumption in (13) is only for the observations, whereas the genes have interactions at the state level, following the underlying Boolean network, so that they cannot be considered independent. Due to the Markov property in (1), $p\left (\mathcal {X}|p_{2:m},S=s\right)$ in (10) can be factored as

14 $$ \begin{aligned} P(\mathcal{X} | p_{2:m}, S&=s) = \\ P(\mathbf{X}_{1}| S&=s) \prod_{k=1}^{m-1} P(\mathbf{X}_{k+1} | \mathbf{X}_{k}, p_{k+1}, S=s), \end{aligned}  $$

where *P*(**X**_*k*+1_|**X**_*k*_,*p*_*k*+1_,*S*=*s*) is the probability of transitioning from state **X**_*k*_ at time *k* to state **X**_*k*+1_ at time *k*+1, given the perturbation probability *p*_*k*+1_, in the class *S*=*s*, and *P*(**X**_1_|*S*=*s*) is the probability of the first state **X**_1_ in the class *S*=*s*. Let **x**^*i*^ denote the *n*×1 Boolean vector of the state *i*, for *i*=1,2,⋯,2^*n*^. Given the perturbation probability *p*_*k*+1_, the conditional transition probability matrix (TPM) at time *k*+1, which is a 2^*n*^×2^*n*^ matrix, in the class *S*=*s* can be derived from (1) as

15 $$ \begin{aligned} \mathbf{A}^{(s)}_{i,j}(k+1) &= P\left(\mathbf{X}_{k+1}=\mathbf{x}^{j} | \mathbf{X}_{k}=\mathbf{x}^{i}, p_{k+1}, S=s\right)\\ &= p_{k+1}^{\mathbf{d}\left(\mathbf{x}^{j},\mathbf{f}_{s}\left(\mathbf{x}^{i}\right)\right)}(1-p_{k+1})^{n-\mathbf{d}\left(\mathbf{x}^{j},\mathbf{f}_{s}\left(\mathbf{x}^{i}\right)\right)}, \end{aligned}  $$

where **d**(**x**^*j*^,**f**_*s*_(**x**^*i*^)) is the Hamming distance between the two Boolean vectors **x**^*j*^ and **f**_*s*_(**x**^*i*^).

For obtaining $p(\mathcal {Y} | S=s)$, we need to integrate out the joint distribution $p(\mathcal {Y},\mathcal {X},\Theta _{s} | S=s)$ in (10) with respect to *Θ*_*s*_ and $\mathcal {X}$:

16 $$ {{\begin{aligned} p\left(\mathcal{Y} | S=s\right) &= \sum_{\mathcal{X}} \int_{\Theta_{s}} p\left(\mathcal{Y},\mathcal{X},\Theta_{s} | S=s\right) \\ &= \sum_{\mathcal{X}} \left\lbrace P(\mathbf{X}_{1} | S=s) \prod_{k=1}^{m-1} \int_{p_{k+1}} g(p_{k+1}) p_{k+1}^{\mathbf{d}\left(\mathbf{X}_{k+1},\mathbf{f}_{s}\left(\mathbf{X}_{k}\right)\right)} \right. \\ & \quad\times (1-p_{k+1})^{n-\mathbf{d}\left(\mathbf{X}_{k+1},\mathbf{f}_{s}\left(\mathbf{X}_{k}\right)\right)} d p_{k+1} \\ & \quad\times \prod_{k=1}^{m} \prod_{j=1}^{n} \int_{\theta_{j}(k)} \int_{\lambda_{j}(k)} p\left(y_{j}(k) | \theta_{j}(k), \lambda_{j}(k)\right) \\ & \quad\left. \times p\left(\theta_{j}(k) | \lambda_{j}(k),x_{j}(k)\right) ~ p\left(\lambda_{j}(k)\right) ~ d\theta_{j}(k) d \lambda_{j}(k) \right\rbrace. \end{aligned}}}  $$

Fortunately, as the priors are conjugate, we can analytically solve the integrals in (16). We will use the following lemmas to do so.

##### **Lemma 1**

Let **P**=(0 1) be the domain of *p*_*k*+1_. The following equation holds:

17 $$ {}\begin{aligned} K_{1} &\triangleq \int_{\mathbf{P}} g(p_{k+1}) p_{k+1}^{\mathbf{d}(\mathbf{X}_{k+1},\mathbf{f}_{s}(\mathbf{X}_{k}))} \\ & \quad \times (1-p_{k+1})^{n-\mathbf{d}(\mathbf{X}_{k+1},\mathbf{f}_{s}(\mathbf{X}_{k}))} d p_{k+1} \\ &= \frac{ \Gamma(\mathbf{d}(\mathbf{X}_{k+1},\mathbf{f}_{s}(\mathbf{X}_{k})) + a) \Gamma(n - \mathbf{d}(\mathbf{X}_{k+1},\mathbf{f}_{s}(\mathbf{X}_{k})) + b)}{\Gamma(a) \Gamma(b) \Gamma(a+b+n)\Gamma(a+b)^{-1}}. \end{aligned}  $$

##### *Proof*

See Appendix 1 in Additional file 1. □

We assume that the observations $\mathcal {Y}$occur in the steady state. As a result, in (16), *P*(**X**_1_|*S*=*s*) is the steady-state probability of the state **X**_1_ in the class *S*=*s*, for *s*=0,1. The following lemma gives the steady-state distribution.

##### **Lemma 2**

Let $\pi ^{(s)}_{i}=P\left (\mathbf {X}_{1}=\mathbf {x}^{i} | S=s\right)$ denote the steady-state probability of the *i*-th state in the class *S*=*s*, and $\pi ^{(s)}=\left [\pi ^{(s)}_{1},\cdots,\pi ^{(s)}_{2^{n}}\right ]$ be the 1×2^*n*^ vector of the steady-state distribution. Then *π*^(*s*)^ can be calculated from

18 $$ \pi^{(s)} = \pi^{(s)} \mathbf{M}^{(s)}, \quad \sum_{i=1}^{2^{n}} \pi_{i}^{(s)}=1,  $$

where **M**^(*s*)^ is the transition probability matrix of the class *S*=*s* with the entries

19 $${} \mathbf{M}^{(s)}_{i,j} = \frac{ \Gamma\left(\mathbf{d}\left(\mathbf{x}^{j},\mathbf{f}_{s}\left(\mathbf{x}^{i}\right)\right) + a\right) \Gamma\left(n - \mathbf{d}\left(\mathbf{x}^{j},\mathbf{f}_{s}\left(\mathbf{x}^{i}\right)\right) + b\right)}{\Gamma(a) \Gamma(b) \Gamma(a+b+n) \Gamma(a+b)^{-1}}.  $$

##### *Proof*

See Appendix 2 in Additional file 1. □

##### **Lemma 3**

Let **Ω**=(−*∞* *∞*) and **Λ**=(0 *∞*) be the domains of *θ*_*j*_(*k*) and *λ*_*j*_(*k*), respectively, for *j*=1,⋯,*n*, and *k*=1,⋯,*m*. The following equation holds:

20 $$ \begin{aligned} K_{2} & \triangleq \int_{\mathbf{\Omega}} \int_{\mathbf{\Lambda}} p(y_{j}(k) | \theta_{j}(k), \lambda_{j}(k)) \\ & \quad \times p(\theta_{j}(k) | \lambda_{j}(k),x_{j}(k)) ~ p(\lambda_{j}(k)) ~ d\theta_{j}(k) d \lambda_{j}(k) \\ & =\frac{1}{(2\pi)^{\frac{1}{2}}} \left(\frac{\kappa_{0}}{\kappa_{1}}\right)^{\frac{1}{2}} \frac{\Gamma(\alpha_{1})}{\Gamma(\alpha_{0})} \frac{\beta_{0}^{\alpha_{0}}}{\beta_{1}^{\alpha_{1}}}, \end{aligned}  $$

where

21 $$ \begin{aligned} \kappa_{1} &= \kappa_{0} + 1, \\ \alpha_{1} &= \alpha_{0}+\frac{1}{2}, \\ \beta_{1} &= \beta_{0} + \frac{\kappa_{0} \left(y_{j}(k)-\mu_{0} - \delta_{0} x_{j}(k)\right)^{2}}{2(\kappa_{0} + 1)}. \end{aligned}  $$

##### *Proof*

See Appendix 3 in Additional file 1. □

#### Summing out $\mathcal {X}$

Using (16), (17), (19), and (20), we have

22 $$ {{\begin{aligned} p(\mathcal{Y}|S=s) &= \sum_{\mathbf{X}_{1}} \cdots \sum_{\mathbf{X}_{m}} \left\lbrace{\vphantom{\left. \quad \times \prod_{k=1}^{m} \prod_{j=1}^{n} \frac{1}{(2\pi)^{\frac{1}{2}}} \left(\frac{\kappa_{0}}{\kappa_{1}}\right)^{\frac{1}{2}} \frac{\Gamma(\alpha_{1})}{\Gamma(\alpha_{0})} \frac{\beta_{0}^{\alpha_{0}}}{\beta_{1}^{\alpha_{1}}} \right\rbrace}} \pi_{\mathbf{X}_{1}}^{(s)} \prod_{k=1}^{m-1} \frac{ \Gamma(\mathbf{d}(\mathbf{X}_{k+1},\mathbf{f}_{s}(\mathbf{X}_{k})) + a) \Gamma(n - \mathbf{d}(\mathbf{X}_{k+1},\mathbf{f}_{s}(\mathbf{X}_{k})) + b)}{\Gamma(a) \Gamma(b) \Gamma(a+b+n) \Gamma(a+b)^{-1}} \right.\\ &\left. \quad \times \prod_{k=1}^{m} \prod_{j=1}^{n} \frac{1}{(2\pi)^{\frac{1}{2}}} \left(\frac{\kappa_{0}}{\kappa_{1}}\right)^{\frac{1}{2}} \frac{\Gamma(\alpha_{1})}{\Gamma(\alpha_{0})} \frac{\beta_{0}^{\alpha_{0}}}{\beta_{1}^{\alpha_{1}}} \right\rbrace. \end{aligned}}}  $$

We use the sum-product algorithm [19] to efficiently compute the summation in (22). Define the 2^*n*^×1 vector *Φ*(*k*) by

23 $${} \begin{aligned} \Phi_{i}(k) &= \left(\frac{ \beta_{0}^{\alpha_{0}}}{(2\pi)^{\frac{1}{2}}} \left(\frac{\kappa_{0}}{\kappa_{0}+1}\right)^{\frac{1}{2}} \frac{\Gamma\left(\alpha_{0}+\frac{1}{2}\right)}{\Gamma(\alpha_{0})}\right)^{n} \\ & \quad \times \prod_{j=1}^{n} \left[\beta_{0} + \frac{\kappa_{0}\left(y_{j}(k)-\mu_{0}-\delta_{0}\mathbf{x}^{i}_{j}\right)^{2}}{2(\kappa_{0}+1)} \right]^{-\left(\alpha_{0}+\frac{1}{2}\right)}, \end{aligned}  $$

for *i*=1,⋯,2^*n*^, where $\mathbf {x}^{i}_{j}$ is the *j*-th entry of the Boolean state **x**^*i*^. We define an auxiliary 2^*n*^×1 vector *Π*^(*s*)^(*k*) at the time *k*, for *k*=1,⋯,*m*, which is initialized and updated as follows:

24 $$\begin{array}{@{}rcl@{}} \Pi^{(s)}(1) &=& \left({\pi}^{(s)}\right)^{T} \circ \Phi(1),  \\ \Pi^{(s)}(k+1) &=& \left[{\mathbf{M}^{(s)}}^{T} \Pi^{(s)}(k)\right] \circ \Phi(k+1), \end{array} $$

for *k*=1,⋯,*m*−1, where *T* denotes the transpose, and ∘ is the Hadamard product. Once we have calculated *Π*^(*s*)^(*m*), the summation of (22) is equal to the *l*_1_ norm ∥*Π*^(*s*)^(*m*)∥_1_, which is the summation of the all 2^*n*^ entries of *Π*^(*s*)^(*m*). Therefore, (22) can be written as

25 $$ p(\mathcal{Y}|S=s) = \parallel \Pi^{(s)}(m) \parallel_{1}.  $$

## Results and discussion

**Fig. 1:**
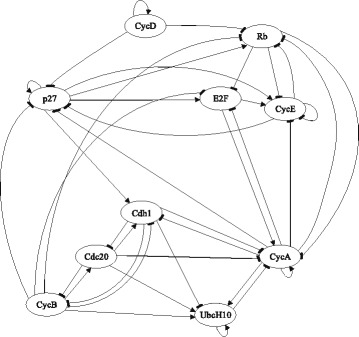
Mammalian cell-cycle gene regulatory network

**Fig. 2:**
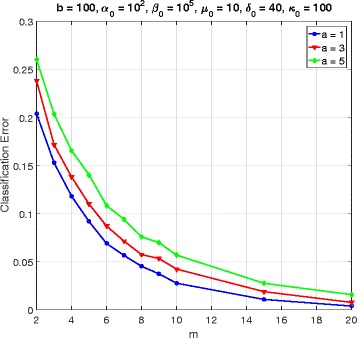
Classifier error versus *m* in cell-cycle network

**Fig. 3:**
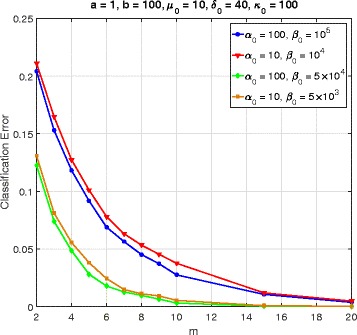
Classifier error versus *m* in cell-cycle network

**Fig. 4:**
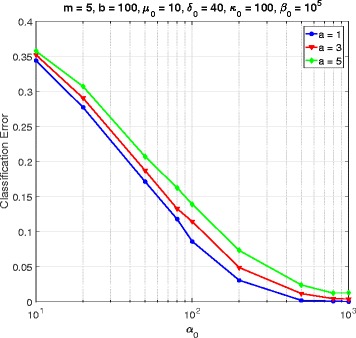
Classifier error versus *α*_0_ in cell-cycle network

**Fig. 5:**
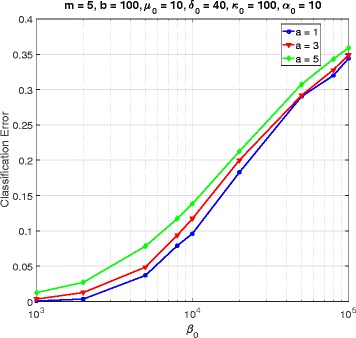
Classifier error versus *β*_0_ in cell-cycle network

**Fig. 6:**
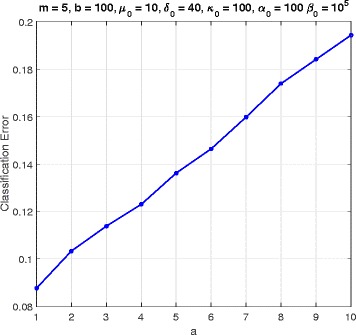
Classifier error versus *a* in cell-cycle network

**Fig. 7:**
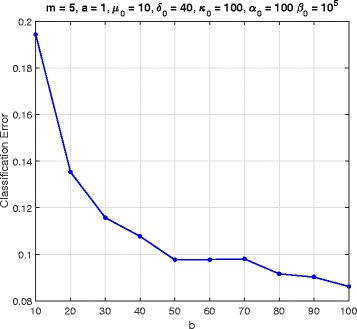
Classifier error versus *b* in cell-cycle network

**Fig. 8:**
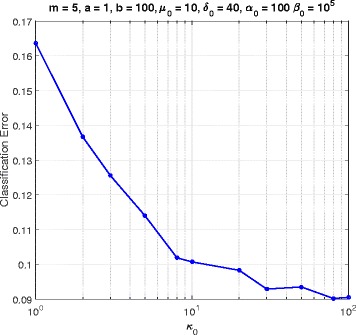
Classifier error versus *κ*_0_ in cell-cycle network

**Fig. 9:**
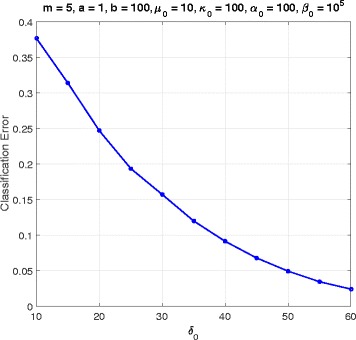
Classifier error versus *δ*_0_ in cell-cycle network

In this section, we consider a mammalian cell-cycle gene regulatory network [17], depicted in Fig. 1, for evaluating our proposed trajectory-based IBR classifier. This GRN consists of *n*=10 genes, whose interactions are shown in Fig. 1. The Boolean functions of the corresponding Boolean network for this GRN are given in Table 1 [17]. We define class *S*=0 as the wild-type class, whose network function **f**_0_ is in Table 1. The value of the gene CycD is determined by extracellular signals, and we assume it is *f*_1_=0. According to [17], one mutated case which leads to cancer is when the gene p27 in the network is shut down and cannot be activated by its regulating genes, that is, *f*_3_=0. Therefore, we define class *S*=1 as the mutated class with the network function **f**_1_, which is the same as Table 1 with *f*_3_=0. We analyze the effects of the hyperparameters and *m* on the classification error in Figs. 2, 3, 4, 5, 6, 7, 8 and 9. In the simulations, we have set *a*_*c*_=*b*_*c*_=10 and *μ*_0_=10. Therefore, the prior probability *c* of the class *S*=0 will have the mean value E[ *c*]=0.5.

**Table 1:** Definitions of Boolean functions for the wild-type mammalian cell-cycle BN with 10 genes

Order	Gene	Regulating function
*x*_1_	*CycD*	*f*_1_= Extracellular signals
*x*_2_	*Rb*	$f_{2}=(\overline {CycD} \wedge \overline {CycE} \wedge \overline { CycA} \wedge \overline {CycB}) \vee (p27 \wedge \overline {CycD} \wedge \overline {CycB})$
*x*_3_	*p*27	$f_{3}=(\overline {CycD} \wedge \overline {CycE} \wedge \overline {CycA} \wedge \overline {CycB}) \vee (p27 \wedge \overline {(CycE \wedge CycA)} \wedge \overline {CycD} \wedge \overline {CycB})$
*x*_4_	*E*2*F*	$f_{4}=(\overline {Rb} \wedge \overline {CycA} \wedge \overline { CycB}) \vee (p27 \wedge \overline {Rb} \wedge \overline {CycB})$
*x*_5_	*CycE*	$f_{5}=(E2F \wedge \overline {Rb})$
*x*_6_	*CycA*	$f_{6}=(E2F \wedge \overline {Rb} \wedge \overline {Cdc20} \wedge \overline {(Cdh1 \wedge UbcH10)}) \vee (CycA \wedge \overline {Rb} \wedge \overline {Cdc20} \wedge \overline {(Cdh1 \wedge UbcH10)})$
*x*_7_	*Cdc*20	*f*_7_=*CycB*
*x*_8_	*Cdh*1	$f_{8}=(\overline {CycA} \wedge \overline {CycB}) \vee Cdc20 \vee (p27 \wedge \overline {CycB})$
*x*_9_	*UbcH*10	$f_{9}=\overline {Cdh1} \vee (Cdh1 \wedge UbcH10 \wedge (Cdc20 \vee CycA \vee CycB))$
*x*_10_	*CycB*	$f_{10}=(\overline {Cdc20} \wedge \overline {Cdh1})$

Figure 2 shows the classification error versus *m* for *a*=1,3,5, when the values of the other hyperparameters are *b*=100, *α*_0_=100, *β*_0_=10^4^, *δ*_0_=40, and *κ*_0_=100. We see that the classification error decreases by increasing *m* and converges to zero. This means that for long enough trajectories we can have perfect classification. The value of the hyperparameter *a* determines the amount of uncertainty for the gene perturbation probability *p*_*k*_ at time *k*. From a biological perspective, we know that *p*_*k*_ should be small. As a result, we have chosen *b*=100, and *a*=1,3,5, leading to the mean values of *p*_*k*_, respectively, as $\mathrm {E}\left [p_{k}\right ]=\frac {a}{a+b}\approx 0.01, 0.03, 0.05$. For a given *b*, the bigger value of *a* allows a wider range of *p*_*k*_, which results in higher classification error.

Figure 3 represents the classification error versus *m* for different values of *α*_0_ and *β*_0_, when *a*=1, *b*=100, *δ*_0_=40, and *κ*=100. As the precision (inverse of variance) has a Gamma(*α*_0_,*β*_0_) distribution, its mean and variance are equal to $\mathrm {E}\left [\lambda \right ]=\frac {\alpha _{0}}{\beta _{0}}$ and $\text {Var}\left [\lambda \right ]=\frac {\alpha _{0}}{\beta _{0}^{2}}=\frac {\mathrm {E}[\lambda ]}{\beta _{0}}$. Figure 3 shows the error curves for the two cases $\frac {\alpha _{0}}{\beta _{0}}=10^{-3}$ and 2×10^−3^, each having a different value of *β*_0_, leading to a different variance of *λ*. As such, we can see the effects of both the mean and variance of the precision *λ*. Whenever $\frac {\alpha _{0}}{\beta _{0}}$ increases, there is lower variance in the outputs, which results in lower errors. We also notice from Fig. 3 that for a given value of $\frac {\alpha _{0}}{\beta _{0}}$, increasing *β*_0_ decreases the error, the reason being that increased *β*_0_ yields lower variance for the precision.

Figure 4 plots the error versus *α*_0_ for *a*=1,3,5, when *m*=5, *b*=100, *δ*_0_=40, *κ*_0_=100, and *β*_0_=10^5^. For all values of *a*, the classification error is a decreasing function of *α*_0_. Similarly, Fig. 5 gives the error versus *β*_0_ for *a*=1,3,5, when *α*_0_=10 and the other hyperparameters are the same as in Fig. 4. Figure 5 shows an increasing trend of classification error as *β*_0_ grows.

Figure 6 shows the error as a function of *a*, when *m*=5, *b*=100, *δ*_0_=40, *κ*_0_=100, *α*_0_=100, and *β*_0_=10^5^. When *a* grows, the uncertainty of the perturbation probability grows as well. The error is an increasing function of *a*. Similarly, Fig. 7 is for error versus *b*, when *a*=1, and the others are the same as in Fig. 6. In Fig. 7 the classification error is decreasing as *b* increases.

Figure 8 illustrates the error versus *κ*_0_, for *m*=5, *a*=1, *b*=100, *δ*_0_=40, *α*_0_=100, and *β*_0_=10^5^. From (5), the hyperparameter *κ*_0_ controls the variance of the mean parameters *θ*_*j*_(*k*). When *κ*_0_ increases, the uncertainty of *θ*_*j*_(*k*) is reduced and its density peaks at *μ*_0_ and *μ*_0_+*δ*_0_ for the unexpressed and expressed states, respectively. Consequently, we expect better error rates for higher *κ*_0_. Accordingly, in Fig. 8 the classification error is a decreasing function of *κ*_0_.

Figure 9 illustrates the error versus *δ*_0_, the expression coefficient, for *m*=5, *a*=1, *b*=100, *κ*_0_=100, *α*_0_=100, and *β*_0_=10^5^. Having larger *δ*_0_ means that the mean values of data for each gene in the unexpressed and expressed cases are well separated, which leads to lower classification error. As expected, in Fig. 9 the error is decreasing as *δ*_0_ gets larger.

## Conclusions

In this paper, we propose a trajectory-based intrinsically Bayesian robust classifier for classification of single-cell gene-expression trajectories. We assume that the expressions of the *n* genes, whose interactions are known in terms of a Boolean network, are observed in *m* consecutive time points, for both the wild-type class (*S*=0) and mutated class (*S*=1). As the parameters have uncertainty, we assign priors for them. We assume a beta distribution as a prior for both the probability of the class *S*=0 and the gene perturbation probabilities at each time. We assume a normal-gamma distribution for the mean and precision of the expressions at each time and for each gene, given the underlying states. As such, we derive closed-form solutions for the effective class-conditional densities of the trajectories in each class, by which we define the IBR classifier. The performance of the IBR classifier is evaluated in a cell-cycle gene regulatory network with 10 genes, for which we know the Boolean networks of the two classes. We have analyzed the effects of *m* and all the hyperparameters on the classification error.

In this paper we have assumed the form of the prior distributions, including the hyperparameter values. Once this is done, the analysis is mathematical: derive the effective class-conditional densities. As is generally the case with Bayesian methods, two issues arise: how are the priors developed and how robust are the methods to the prior assumptions? Both issues have been extensively studied in regard to the OBC, of which the IBR classifier is the special case in which there is no update to a posterior.

In [9] robustness of the OBC relative to various modeling assumptions has been investigated, including false assumptions on the covariance structure and falsely assuming Gaussianity. In [20], similar robustness issues have been considered for the MMSE error estimator, which is required for defining the OBC.

Regarding prior construction, the question is how to transform existing scientific knowledge into a prior distribution. Because the IBR classifier and other IBR filtering methods involve prior distributions on the underlying scientific model, for instance, the stochastic process (power spectra) in Wiener filtering and the feature-label distribution in classification, uncertainty arises from insufficient scientific knowledge, the prior characterizes that uncertainty, and, in effect, constrains the optimization relative to that uncertainty. For IBR/OBC classification in the context of uncertain gene regulatory networks, prior construction methods based on constrained optimization have been developed for both Gaussian [21] and discrete [22] gene regulatory networks under the assumption of static observations. Future work involves extending these prior-construction methods to trajectories, which should be possible, albeit, with more difficult mathematical analysis and substantially more computation.

Another issue to be addressed in future work is extending the trajectory-based IBR classifier to optimal Bayesian classification, which will require analytic representation of the effective class-conditional densities relative to a posterior distribution derived from the prior distribution utilizing sample data.

## Additional file

Additional file 1 Appendices. This additional file contains the appendices for the proofs of the lemmas 1, 2, and 3. (PDF 33 kb)

## Abbreviations

BN: Boolean network
BNp: Boolean network with perturbation
BOA: Basin of attraction
BPOBDS: Bayesian partially observed Boolean dynamical system
EM: Expectation maximization
GRN: Gene regulatory network
IBR: Intrinsically Bayesian robust
MMSE: Minimum mean square error
MSE: Mean square error
OBC: Optimal Bayesian classifier
TPM: Transition probability matrix
