# Supplementary material for: Intrinsically Bayesian robust classifier for single-cell gene expression trajectories in gene regulatory networks
Source: BMC Syst Biol. 2018 Mar 21;12(Suppl 3):23. doi: 10.1186/s12918-018-0549-y (PMC5872504; doi:10.1186/s12918-018-0549-y)
Supplement: Supplementary file 1 — Appendices. This additional file contains the appendices for the proofs of the lemmas 1, 2, and 3. (PDF 33 kb) [file 12918_2018_549_MOESM1_ESM.pdf]

Additional file 1

APPENDIX 1:

Proof of lemma 1: Since $g(p_{k+1})$ is a valid beta probability density, as in (2), its integration with respect to p_{k+1} will be one:

$$\int_{\mathbf{P}} g(p_{k+1})dp_{k+1} = \int_{\mathbf{P}} \frac{\Gamma(a+b)}{\Gamma(a)\Gamma(b)} p_{k+1}^{a-1}(1-p_{k+1})^{b-1}dp_{k+1} = 1. \tag{A1}$$

Hence,

$$\int_{\mathbf{P}} p_{k+1}^{a-1}(1-p_{k+1})^{b-1}dp_{k+1} = \frac{\Gamma(a)\Gamma(b)}{\Gamma(a+b)}. \tag{A2}$$

After replacing $g(p_{k+1})$ in (17),

$$K_1 = \frac{\Gamma(a+b)}{\Gamma(a)\Gamma(b)} \int_{\mathbf{P}} p_{k+1}^{\mathbf{d}(\mathbf{X}_{k+1},\mathbf{f}_s(\mathbf{X}_k))+a-1} \times (1-p_{k+1})^{n-\mathbf{d}(\mathbf{X}_{k+1},\mathbf{f}_s(\mathbf{X}_k))+b-1}dp_{k+1}. \tag{A3}$$

Using (A2) and (A3), K_1 is derived as in (17).

APPENDIX 2:

Proof of lemma 2: It is well-known that the steady-state distribution of a time-homogeneous TPM is obtained from (18). The conditional TPM $\mathbf{A}^{(\mathbf{s})}(k+1)$ in (15) is time-inhomogeneous, since each time has its own perturbation probability p_{k+1}. Since the prior distribution of p_{k+1} in (2) is the same for every k, integrating the conditional TPM $\mathbf{A}^{(\mathbf{s})}(k+1)$, for every k, over the prior distribution of p_{k+1} yields a time-homogeneous TPM with the (i,j)-th entry as

$$\mathbf{M}_{i,j}^{(s)} = \int_{\mathbf{P}} \mathbf{A}_{i,j}^{(s)}(k+1)g(p_{k+1})dp_{k+1} = \int_{\mathbf{P}} g(p_{k+1})p_{k+1}^{\mathbf{d}(\mathbf{x}^j,\mathbf{f}_s(\mathbf{x}^i))}(1-p_{k+1})^{n-\mathbf{d}(\mathbf{x}^j,\mathbf{f}_s(\mathbf{x}^i))}dp_{k+1}. \tag{A4}$$

Lemma 1 and (A4) result in (19).

APPENDIX 3:

Proof of lemma 3: From (5), the normal-gamma prior for $\theta_j(k)$ and $\lambda_j(k)$ is

$$\begin{aligned} p(\theta_j(k), \lambda_j(k)|x_j(k)) &= p(\theta_j(k)|\lambda_j(k), x_j(k))\ p(\lambda_j(k)) \\ &= \frac{1}{Z_0}\lambda_j(k)^{\alpha_0-\frac{1}{2}} \exp\left(-\frac{\lambda_j(k)}{2}\left[\kappa_0(\theta_j(k)-\mu_j(k))^2+2\beta_0\right]\right), \end{aligned} \tag{A5}$$

where

$$Z_0 = \left(\frac{2\pi}{\kappa_0}\right)^{\frac{1}{2}} \frac{\Gamma(\alpha_0)}{\beta_0^{\alpha_0}}. \tag{A6}$$

The likelihood from (4) is

$$p(y_j(k)|\theta_j(k), \lambda_j(k)) = \frac{1}{(2\pi)^{\frac{1}{2}}}\lambda_j(k)^{\frac{1}{2}} \exp\left(-\frac{\lambda_j(k)}{2}(y_j(k)-\theta_j(k))^2\right).$$

Therefore, for the posterior,

$$\begin{aligned} &p(\theta_j(k), \lambda_j(k)|y_j(k), x_j(k)) \propto p(y_j(k)|\theta_j(k), \lambda_j(k))p(\theta_j(k), \lambda_j(k)|x_j(k)) \\ &\propto \lambda_j(k)^{\alpha_0} \exp\left(-\frac{\lambda_j(k)}{2}\left[\kappa_0(\theta_j(k)-\mu_j(k))^2+2\beta_0+(y_j(k)-\theta_j(k))^2\right]\right) \\ &\propto \lambda_j(k)^{\alpha_1-\frac{1}{2}} \exp\left(-\frac{\lambda_j(k)}{2}\left[\kappa_1(\theta_j(k)-\eta_j(k))^2+2\beta_1\right]\right), \end{aligned}$$

where κ_1, α_1, and β_1 are given in (21), and $\eta_j(k)$ is defined by

$$\eta_j(k) = \frac{\kappa_0\mu_j(k)+y_j(k)}{\kappa_0+1}. \tag{A7}$$

Comparing (A7) with (A5), we see that the posterior also has the following normal-gamma density:

$$\begin{aligned} &p(\theta_j(k), \lambda_j(k)|y_j(k), x_j(k)) = \\ &\frac{1}{Z_1}\lambda_j(k)^{\alpha_1-\frac{1}{2}} \exp\left(-\frac{\lambda_j(k)}{2}\left[\kappa_1(\theta_j(k)-\eta_j(k))^2+2\beta_1\right]\right), \end{aligned} \tag{A8}$$

where

$$Z_1 = \left(\frac{2\pi}{\kappa_1}\right)^{\frac{1}{2}} \frac{\Gamma(\alpha_1)}{\beta_1^{\alpha_1}}. \tag{A9}$$

Since the posterior density in (A8) integrates to 1,

$$\int_{\mathbf{\Omega}}\int_{\mathbf{\Lambda}} \lambda_j(k)^{\alpha_1-\frac{1}{2}} \exp\left(-\frac{\lambda_j(k)}{2}\left[\kappa_1(\theta_j(k)-\eta_j(k))^2+2\beta_1\right]\right) d\theta_j(k)d\lambda_j(k) = Z_1.$$

Finally, K_2 in (20) can be written as

$$\begin{aligned} K_2 &= \frac{1}{(2\pi)^{\frac{1}{2}}} \frac{1}{Z_0} \int_{\mathbf{\Omega}} \int_{\mathbf{\Lambda}} \lambda_j(k)^{\alpha_1 - \frac{1}{2}} \exp\left(-\frac{\lambda_j(k)}{2} \left[\kappa_1 (\theta_j(k) - \eta_j(k))^2 + 2\beta_1 \right] \right) d\theta_j(k) d\lambda_j(k) \\ &= \frac{1}{(2\pi)^{\frac{1}{2}}} \frac{Z_1}{Z_0} = \frac{1}{(2\pi)^{\frac{1}{2}}} \left(\frac{\kappa_0}{\kappa_1} \right)^{\frac{1}{2}} \frac{\Gamma(\alpha_1)}{\Gamma(\alpha_0)} \frac{\beta_0^{\alpha_0}}{\beta_1^{\alpha_1}}, \end{aligned}$$

which finishes the proof.
